# Neutrosophic Cost Pattern of Inventory System with Novel Demand Incorporating Deterioration and Discount on Defective Items Using Particle Swarm Algorithm

**DOI:** 10.1155/2022/7683417

**Published:** 2022-08-09

**Authors:** G. Durga Bhavani, Fasika Bete Georgise, G. S. Mahapatra, B. Maneckshaw

**Affiliations:** ^1^Department of Mathematics, National Institute of Technology Puducherry, Karaikal 609 609, India; ^2^Department of Industrial Engineering, Faculty of Manufacturing, Institute of Technology (IoT), Hawassa University, Awasa, Ethiopia

## Abstract

The potential to obtain defective or damaged items with non-defective commodities is common to experience at the production unit or when shipping products from one layer to another. This research focuses on the faulty things that retailers receive from suppliers. The retailer has set a restriction on the percentage of defective things, and the retailer receives a discount on the cost of purchasing defective items. The proposed inventory system handles the uncertainty in inventory costs and also considers the demand and deterioration of items with prioritized maximum product life. This work minimizes total inventory cost when demand rate as a function of reliability and power pattern of time under a crisp and triangular neutrosophic environment. The inventory system for degrading items considers the predictability and power pattern of time with a reasonable payment delay. The interest charges are applied only after a specific permissible time limit in the proposed inventory system. The neutrosophic number that defines three different kinds of membership functions representing the truth, hesitation, and falseness is applied in the inventory model in handling the uncertainty of the cost pattern. The proposed inventory model is investigated using a particle swarm optimization algorithm, and the results are validated using a numerical example and a sensitivity analysis for various parameters.

## 1. Introduction

In dealing with inventory models, researchers need to give more importance to some key factors, such as deterioration, demand, and reliability. It is obvious that deterioration is a time-dependent factor, and furthermore, it increases with time, resulting in a decrease in the demand for the commodity. When products are stored in warehouses, there are plenty of problems, such as reliability, volume, and deterioration of items, which are different for every commodity. Consequently, the holding costs have a major impact on the value of the quantity stored. Deterioration is a progressive degradation of stored products that results in their devaluation. The rate of deterioration is shallow in durable products such as iron products, steels, toys, consumer electronics, furniture, tools, jewelry, automobiles, sports equipment, and so on. The deterioration rate encounters rapid changes in semi-durable products such as food items, drugs, clothing, cosmetics, and so on. Therefore, the study of the deterioration of items in the inventory systems plays a major role due to different deterioration patterns in the economic order quantity (EOQ) model. Tadikamalla [1] introduced an EOQ model using a gamma distribution to represent the constant, increasing, and decreasing rates of deterioration over time. Alshanbari et al. [2] proposed an inventory model for deteriorating items with two-parameter Weibull distribution. Wang and Lin [3] developed the optimal replenishment strategy with deterioration, market demand, and price changes. An inventory model for deteriorating items with maximum lifetime was proposed by Wang et al. [4]. Skouri et al. [5] proposed an inventory model with shortages, ramp-type demand rate, and time-dependent deterioration rate. Defective items are usually observed in the inventory management system, including the possibility of being defective during shipment from the warehouse to the supplier or retailer's warehouse. Several researchers [6] considered defective rate of items in the inventory system to develop their models. This article presents an inventory model that handles the rate of deterioration in such a way that the maximum useful life of the product is prioritized, and this study also considers the defective rate.

Demand is critical to inventory management, and future inventory cannot be forecast without considering demand. For each item, the demand rate is different. The demand rate for some items rises at the start of the cycle. The demand rate for some items remains constant throughout the cycle, whereas the demand rate for others increases at the end. The demand rate for cooked items such as bread, sweets, cakes, and so on increases at the beginning of the cycle because customers love just-made goods. Due to the expiration date, the demand rate decreases at the end of the cycle for goods such as fish, vegetables, fruits, and so on. On the contrary, others have an increasing demand rate at the end of the cycle, such as household goods such as oil, sugar, milk, and so on, and the demand rate is constant for furniture, electrical goods, etc. Khedlekar and Sukhla [7] developed a dynamic pricing model for logarithmic demand. Smaila and Chukwu [8] introduced an EOQ model with quadratic demand trends and quasi-partial backlogging. Dutta Choudhury et al. [9] presented an inventory model by taking two-component demand. Prasad and Mukherjee [10] developed an inventory model on stock and time-dependent demand. Wu et al. [11] developed an inventory policy for trapezoidal-type demand patterns and maximum lifetime under trade credits. Mahapatra et al. [12] presented an EPQ model with demand and reliability-dependent unit production cost under limited available intuitionistic fuzzy type storage space. Shaikh and Mishra [13] developed an EOQ model for price-sensitive quadratic demand and inflationary conditions. Delay in payment is a common phenomenon in the inventory management system. A delay in payment is offered by the supplier, where the retailer's purchase cost is paid at a later date without an interest charge. Liao et al. [14] proposed an inventory model with items that deteriorate under inflation and allow a delay in payment. Several researchers [15–18] developed their inventory models under the condition of delay in payment. Some researchers used other payment schemes to develop their models. Teng et al. [19] presented an inventory model under a progressive payment strategy. Teng et al. [20] presented an EOQ model under a trade credit financing scheme. This study of inventory management allows a delay in payment for two different situations based on time.

Neutrosophic numbers explain the impreciseness of the systems. In real life, most of the parameters are uncertain, so in this situation, neutrosophic numbers play a crucial role in overcoming uncertainty. Mullai and Surya [21] developed a price-break EOQ model with neutrosophic demand and purchasing cost as triangular neutrosophic numbers. Mariagrazia et al. [22] proposed a supplier selection methodology under uncertainty. Ge and Zhang [23] presented an inventory model under a fuzzy uncertain environment. De et al. [24] developed an inventory model for the non-random uncertain environment using the neutrosophic fuzzy approach. Mariagrazia et al. [25] introduced the fuzzy technique for the supply chain network with quantity discounts. Das and Tripathy [26] investigated some properties of neutrosophic multiset topology. Bonilla-Enriquez et al. [27] proposed a supply chain model taking into account uncertain demand. The present paper considers the cost pattern of the inventory system as a triangular neutrosophic number.

Generally, inventory problems are solved by traditional direct optimization methods, but one of the shortcomings is that they are very often stuck to local optima. To avoid several shortcomings of global optimization, particle swarm optimization (PSO) is very useful in solving problems in the field of inventory control. Biuki et al. [28] presented an inventory problem in optimizing through two hybrid metaheuristics as parallel and series combinations of genetic algorithm and PSO. Alejo-Reyes et al. [29] introduced an inventory model for supplier selection and order quantity allocation by using metaheuristic algorithms. Rau et al. [30] proposed a multiobjective green cyclic inventory routing problem via the discrete multiswarm PSO method. Al-Khazraji et al. [31] applied multiobjective PSO to optimize production inventory control systems. Patne et al. [32] presented a closed-loop supply chain network configuration model using game-theoretic PSO. Dabiri et al. [33] presented a bi-objective inventory routing problem with a step cost function using multiobjective PSO. Manatkar et al. [34] presented an integrated inventory distribution optimization model for multiple products using a novel hybrid multiobjective self-learning PSO method. Srinivasan et al. [35] applied PSO to optimize a mathematical model with defective goods. A multiitem EPQ model with a production capacity restriction using the PSO algorithm was developed by Pirayesh and Poormoaied [36]. Araya-Sassi et al. [37] developed an inventory location problem by using the Lagrangian relaxation algorithm. This article considers the PSO algorithm to find the optimal solution under the neutrosophic uncertainty-based cost metrics of the presented inventory system. A comparison of the contributions is presented in Table 1 to determine the most significant contribution of this work. The contribution of this study is to offer the best cycle duration and reduce the total cost of the inventory system under triangular neutrosophic cost pattern when the customer demand depends on time and reliability. To the best of our knowledge, this work addresses all of the themes stated in Table 1 simultaneously first time, which have never been examined together in the literature before.

Simultaneous evaluation of the preceding assumptions allows us to represent a wide range of real-life situations, resulting in a more realistic inventory model. The remainder of this article is organized as follows. The inventory problem is mathematically formulated in Section 2.  Section 3 develops the model formulation in a neutrosophic environment.  Section 4 contains the proposed inventory model using the PSO algorithm. In Section 5, a numerical example is explained, and then a sensitivity analysis of the optimal inventory policy for the system input parameters is presented, along with some significant managerial insights obtained from the results.  Section 6 concludes with some findings and research directions for the future.

## 2. Mathematical Modeling of the Inventory System

Based on the discussion in Introduction, the following notations are considered to present the inventory system.

### 2.1. Notations

 
*C*_0_: ordering cost per order ($/order).
 
*C*: purchasing cost per item ($/unit).
 
*S*: selling price ($/unit).
 
*C*_*h*_: holding cost per item ($/unit/unit time).
 
*I*_*c*_: rate of interest charged per year in stocks by suppliers.
 
*I*_*e*_: rate of interest earned by investment per year.
 
*θ*(*t*): deterioration rate of items.
 
*m*: maximum life time in years of item, (*m* > 2).
 
*T*_*d*_: supplier permissible delay period.
 
*T*: cycle time in per cycle.
 
*f*: percentage of non-defective items (0.9 ≤ *f* ≤ 1).
 
*r*: reliability of item (0 < *r* < 1).
 
*γ*: reduction percentage of purchasing cost of defective items.
 
*d*: average demand per cycle (*d*=*x*/*T* > 0).
 
*x*: total demand per cycle.
 
*n*: demand pattern index (*n* > 0).

### 2.2. Problem Definition

**Figure 1:**
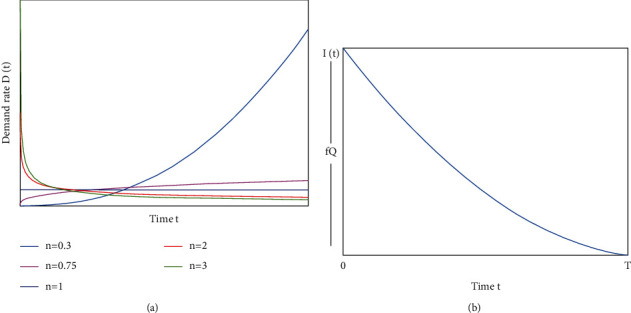
Graphical representation of demand and inventory model.

This paper considers mathematical modeling of the fact that, in reality, the rate of deterioration increases over time, where the maximum lifetime of an item is known to the suppliers. The shortages are not allowed for the proposed inventory system through the maximum life span is considered for the item in each replenishment cycle. Demand is the main factor in inventory modeling, which depends on several factors, but reliability is one of the main characteristics. Therefore, the demand rate of the commodities for this study depends on the reliability and the power pattern of time, i.e., the demand rate in a period of time *t* is defined as *D*(*t*)=xar^*α*^/*n*(*t*^1/*n*−1^/*T*^1/*n*^), where *n* seems to be the demand pattern index, and *n* > 0, and it is worth noting that if *n* > 1, a larger portion of demand occurs towards the beginning of the period. If *n*=1, the demand rate remains constant throughout the inventory cycle. However, if *n* < 1, a large portion of the demand comes near the end of the cycle, as shown in Figure 1(a).

The deterioration rate of the inventory system increases with time. It depends on the maximum shelf life of the product in the inventory system, so the deterioration rate of this model is *θ*(*t*) = 1/*m* − *t*, 0 ≤ *t* ≤ *T* < *m*. The supplier offers a reduction rate on the purchase cost of damaged or defective products of this model to the buyer. The inventory system considers a permissible delay in payments, where the credit period is less than or equal to *T*_*d*_ during which the buyer does not need to pay any interest to the retailer, where the interest is applied beyond the period *T*_*d*_. The costs related to the inventory system are imprecise. Therefore, to represent the impreciseness of the inventory costs, we consider the inventory cost parameters as triangular neutrosophic numbers instead of a fixed crisp value. The retailer separates defective and non-defective items by screening items received from the supplier. The retailer fixed the rate of the range of non-defective items which is 0.9 ≤ *f* ≤ 1, which means that the retailer does not accept stock that contains 10% or above defective items. The retailer also receives a discount on the purchase cost of defective items.

### 2.3. Mathematical Formulation

Based on these considerations, the inventory system (shown in Figure 1(b)) can be represented in the time interval [0, *T*], using the differential equation as given below:

(1) $$\begin{array}{c} \frac{\text{dI}\left(t\right)}{\text{dt}}+\frac{1}{m-t}I\left(t\right)=-\frac{{\text{dar}}^{\alpha }t^{1/n-1}}{nT^{1/n-1}},\text{for }0\leq t\leq T, \end{array}$$

subject to the boundary conditions: *I*(*T*)=0 and *I*(0)=*fQ*, where *Q* is the initial inventory for each cycle.

**Lemma 1 .**

*The deterioration rateθ*(*t*)*is decreasing with maximum life time of the product and increases with time.*

**Proof**

The deterioration rate of the proposed inventory system is *θ*(*t*)=1/*m* − *t*, where 0 ≤ *t* ≤ *T* < *m*.

*dθ*(*t*)/dm=−1/(*m* − *t*)^2^ < 0 for 0 ≤ *t* ≤ *T* < *m*.

Therefore, the deterioration rate *θ*(*t*) decreases with the maximum shelf life of the product.

*dθ*(*t*)/dt=1/(*m* − *t*)^2^ > 0 for 0 ≤ *t* ≤ *T* < *m*.

Therefore, the deterioration rate *θ*(*t*) decreases with the maximum shelf life of the product and increases with time.

Hence the proof.

**Lemma 2 .**

*The demand rate D(t) is decreasing with time forn* > 1*, increasing with time for*0 < *n* < 1, *and constant with time forn*=1.

**Proof**

The demand rate of the given model is *D*(*t*)=dar^*α*^/*n*(*t*/*T*)^1/*n*−1^. For time *t*, dD(*t*)/dt=dar^*α*^(1 − *n*)/*n*^2^*T*^1/*n*−1^(*t*)^1/*n*−2^ > 0, for 0 < *n* < 1 and for all *t*, which shows that demand rate increasing with time.

For the time *t*, *dD*(*t*)/dt=dar^*α*^(1 − *n*)/*n*^2^*T*^1/*n*−1^(*t*)^1/*n*−2^=0, for *n*=1 and for all *t*, which shows that demand rate is constant with time.

For the time *t*, dD(*t*)/dt=dar^*α*^(1 − *n*)/*n*^2^*T*^1/*n*−1^(*t*)^1/*n*−2^ < 0, for *n* > 1 and for all *t*, which shows that demand rate is decreasing with time.

Hence the proof.

Using these boundary conditions, the solution of differential (1) of the proposed inventory model during the time interval 0 ≤ *t* ≤ *T* is as follows:

(2) $$\begin{array}{c} I\left(t\right)=\frac{{\text{dar}}^{\alpha }\left(m-t\right)}{{\text{mT}}^{1/n-1}}\left[\left(T^{1/n}-t^{1/n}\right)+\frac{1}{m\left(n+1\right)}\left(T^{1/n+1}-t^{1/n+1}\right)\right]. \end{array}$$

Again using boundary condition *I*(0)=fQ, we get

(3) $$\begin{array}{c} Q=\frac{{\text{dar}}^{\alpha }T}{f}\left[1+\frac{T}{m\left(n+1\right)}\right]. \end{array}$$

Now, using (2) and (3), the holding cost (HC), the purchasing cost (PC), and the ordering cost (OC) of the proposed inventory model can be obtained as given in the succeeding equations:

(4) $$\begin{array}{c} \text{HC}=C_{h}\int_{0}^{T}I\left(t\right)\text{dt}=\frac{nC_{h}T^{2}{\text{dar}}^{\alpha }}{m}\left[\frac{m}{n+1}+\frac{T}{2\left(2n+1\right)}-\frac{T^{2}}{2m\left(3n+1\right)}\right]. \end{array}$$

The purchase cost of the inventory system consider per item and required to purchase the item for inventory.

(5) $$\begin{array}{c} \text{PC}=\text{CQ}=\frac{C{\text{dar}}^{\alpha }T}{f}\left[1+\frac{T}{m\left(n+1\right)}\right]. \end{array}$$

The cost of ordering is fixed for each cycle of unit inventory model.

(6) $$\begin{array}{c} OC=C_{0}. \end{array}$$

The supplier gives a discount *γ* to the purchasing cost of damaged or defective items of the inventory. The discount amount obtained by purchasing defective items is

(7) $$\begin{array}{c} \gamma C\left(1-f\right)Q=\gamma C\left(1-f\right)\frac{{\text{dar}}^{\alpha }T}{f}\left[1+\frac{T}{m\left(n+1\right)}\right]. \end{array}$$

The retailers of this inventory system allow for a conditional delay in payment for the buyer. Therefore, the two cases are arrived at such that the delay period is greater than the cycle time and delay period is less than the cycle time. Now, we discuss the interests due to delayed payments for both cases to obtain the total inventory costs of the following two cases.

**Case 1 .**

Delay period is greater than the cycle time (*T* ≤ *T*_*d*_).

Here, the retailer earns interest per cycle at a return rate *I*_*e*_; if *T* ≤ *T*_*d*_, then the annual interest earned per cycle is given by

(8) $$\begin{array}{c} {\text{IE}}_{1}={\text{SI}}_{e}\left[\int_{0}^{T}D\left(t\right)t\text{dt}+\left(T_{d}-T\right)\int_{0}^{T}D\left(t\right)\text{dt}\right]=\frac{{\text{STI}}_{e}{\text{dar}}^{\alpha }}{\left(n+1\right)}\left[n\left(T_{d}-T\right)+T_{d}\right]. \end{array}$$

The total cost of inventory per unit time in situation *T* ≤ *T*_*d*_ is obtained as follows:

(9) $$\begin{array}{c} TC_{1}=\frac{1}{T}\left(HC+OC+PC-C\gamma \left(1-f\right)Q-IE_{1}\right)\\ =\frac{C_{h}T{\text{dar}}^{\alpha }}{m}\left(\frac{m}{n+1}+\frac{T}{2\left(2n+1\right)}-\frac{T^{2}}{2m\left(3n+1\right)}\right)-\frac{SI_{e}{\text{dar}}^{\alpha }}{\left(n+1\right)}\left(n\left(T_{d}-T\right)+T_{d}\right)\\ +\frac{C_{0}}{T}+\frac{C{\text{dar}}^{\alpha }}{f}\left(1+\frac{T}{m\left(n+1\right)}\right)\left(1-\left(1-f\right)\gamma \right). \end{array}$$

The total inventory cost of the proposed inventory model under the delay period is greater than the cycle time *TC*_1_ which is to be optimized with respect to the optimal cycle *T*^*∗*^ using PSO.

**Case 2 .**

Delay period is less than the cycle time (*T*_*d*_ < *T*).

The interest charged (IC) by the retailer per cycle is obtained by

(10) $$\begin{array}{c} IC=CI_{C}\int_{T_{d}}^{T}I\left(t\right)\text{dt}\\ =\frac{{\text{CTI}}_{C}{\text{dar}}^{\alpha }}{m}\left[\frac{mT}{n+1}+\frac{T^{2}}{2\left(2n+1\right)}-\frac{T^{3}}{2m\left(3n+1\right)}-\frac{T_{d}\left(2m-T_{d}\right)}{2}\left(1+\frac{T}{m\left(n+1\right)}\right)\right.\\ \left.+\frac{nT_{d}^{1/n+1}}{T^{1/n}}\left(\frac{m}{\left(n+1\right)}-\frac{nT_{d}}{\left(2n+1\right)\left(n+1\right)}-\frac{T_{d}^{2}}{m\left(n+1\right)\left(3n+1\right)}\right)\right]. \end{array}$$

The interest earned during the time 0 to *T*_*d*_ is given by

(11) $$\begin{array}{c} IE_{2}=SI_{e}\int_{0}^{T_{d}}D\left(t\right)\text{tdt}=\frac{SI_{e}T_{d}^{1/n+1}{\text{dar}}^{\alpha }}{\left(n+1\right)T^{1/n-1}}. \end{array}$$

In situation *T*_*d*_ < *T*, the total cost of inventory per unit of time (*TC*_2_) is obtained as

(12) $$\begin{array}{c} TC_{2}=\frac{1}{T}\left(HC+PC+OC-C\gamma \left(1\right.-f\left.Q\right)-IE_{2}+IC\right)\\ =\frac{C_{h}T{\text{dar}}^{\alpha }}{m}\left(\frac{m}{n+1}+\frac{T}{2\left(2n+1\right)}-\frac{T^{2}}{2m\left(3n+1\right)}\right)+\frac{C{\text{dar}}^{\alpha }}{f}\left(1+\frac{T}{m\left(n+1\right)}\right)\left(1-\left(1-f\right)\gamma \right)\\ +\frac{C_{0}}{T}+\frac{nCI_{C}{\text{dar}}^{\alpha }}{m}\left[\frac{mT}{n+1}+\frac{T^{2}}{2\left(2n+1\right)}-\frac{T^{3}}{2m\left(3n+1\right)}-\frac{T_{d}\left(2m-T_{d}\right)}{2}\left(1+\frac{T}{m\left(n+1\right)}\right)\right.\\ \left.+\frac{nT_{d}^{1/n+1}}{T^{1/n}}\left(\frac{m}{\left(n+1\right)}-\frac{nT_{d}}{\left(2n+1\right)\left(n+1\right)}-\frac{T_{d}^{2}}{m\left(3n+1\right)\left(n+1\right)}\right)\right]-\frac{SI_{e}T_{d}^{1/n+1}dar^{\alpha }}{\left(n|+|1\right)T^{1/n}}. \end{array}$$

## 3. Proposed Inventory Model under Neutrosophic Environment

Neutrosophic number deals with uncertainties better than fuzzy and intuitionistic fuzzy numbers, since neutrosophic numbers contain truth, hesitant, and falsity membership functions to deal with all types of uncertainties of the parameters. In a real market, the cost parameters are uncertain and contain a dilemma in the decision maker's mind. Thus, this study attempts to manifest the inventory system by introducing a neutrosophic set to represent the different inventory costs and rates of the proposed inventory system. Thus, observe the effect of uncertainty by comparing it with the crisp model. In the proposed inventory model, this paper has considered holding cost (*C*_*h*_), purchase cost (*C*), ordering cost (*C*_0_), and selling price (*S*) as triangular neutrosophic numbers [49]. The representation of triangular neutrosophic numbers $\tilde{C_{h}}$, $\tilde{C}$, $\tilde{C_{0}}$, $\tilde{S}$ is as follows: $\tilde{C_{h}}=\langle \left(h_{1}-\epsilon_{1},h_{1},h_{1}+\epsilon_{2}:\mu \right),\left(h_{2}-\epsilon_{1},h_{2},h_{2}+\epsilon_{2}:\nu \right),\left(h_{3}-\epsilon_{1},h_{3},h_{3}+\epsilon_{2}:\zeta \right)\rangle$, $\tilde{C}=\langle \left(c_{1}-\epsilon_{1},c_{1},c_{1}+\epsilon_{2}:\mu \right),\left(c_{2}-\epsilon_{1},c_{2},c_{2}+\epsilon_{2}:\nu \right),\left(c_{3}-\epsilon_{1},c_{3},c_{3}+\epsilon_{2}:\zeta \right)\rangle$, $\tilde{C_{0}}=\langle \left(p_{1}-\epsilon_{1},p_{1},p_{1}+\epsilon_{2}:\mu \right),\left(p_{2}-\epsilon_{1},p_{2},p_{2}+\epsilon_{2}:\nu \right),\left(p_{3}-\epsilon_{1},p_{3},p_{3}+\epsilon_{2}:\zeta \right)\rangle$, $\tilde{S}=\langle \left(s_{1}-\epsilon_{1},s_{1},s_{1}+\epsilon_{2}:\mu \right),\left(s_{2}-\epsilon_{1},s_{2},s_{2}+\epsilon_{2}:\nu \right),\left(s_{3}-\epsilon_{1},s_{3},s_{3}+\epsilon_{2}:\zeta \right)\rangle$.

Now using the removal area technique for the de-neutrosophic of the neutrosophic numbers to find the optimum inventory costs, are obtained as follows: ${\tilde{C_{h}}}_{\left(D\right)}=h_{1}+h_{2}+h_{3}/3-\epsilon_{1}-\epsilon_{2}/4$, ${\tilde{C}}_{\left(D\right)}=c_{1}+c_{2}+c_{3}/3-\epsilon_{1}-\epsilon_{2}/4$, ${\tilde{C_{0}}}_{\left(D\right)}=p_{1}+p_{2}+p_{3}/3-\epsilon_{1}-\epsilon_{2}/4$, and ${\tilde{S}}_{\left(D\right)}=s_{1}+s_{2}+s_{3}/3-\epsilon_{1}-\epsilon_{2}/4$.

To obtain the total cost in the neutrosophic domain $\left(\tilde{TC_{1}},\tilde{TC_{2}}\right)$, substituting the values of de-neutrosophic values ${\tilde{C_{h}}}_{\left(D\right)}$, ${\tilde{C}}_{\left(D\right)}$, ${\tilde{C_{0}}}_{\left(D\right)}$, and ${\tilde{S}}_{\left(D\right)}$ into the total cost of both cases in (9) and (12), we get

(13) $$\begin{array}{c} \tilde{TC_{1}}=\frac{{\tilde{C}}_{\left(D\right)}{\text{dar}}^{\alpha }}{f}\left(1+\frac{T}{m\left(n+1\right)}\right)\left(1-\left(1-f\right)\gamma \right)-\frac{{\tilde{S}}_{\left(D\right)}I_{e}{\text{dar}}^{\alpha }}{\left(n+1\right)}\left(n\left(T_{d}-T\right)+T_{d}\right)\\ +\frac{{\tilde{C_{0}}}_{\left(D\right)}}{T}+\frac{{\tilde{C_{h}}}_{\left(D\right)}T{\text{dar}}^{\alpha }}{m}\left(\frac{m}{n+1}+\frac{T}{2\left(2n+1\right)}-\frac{T^{2}}{2m\left(3n+1\right)}\right), \end{array}$$

(14) $$\begin{array}{c} \tilde{TC_{2}}=\frac{{\tilde{C}}_{\left(D\right)}{\text{dar}}^{\alpha }}{f}\left(1+\frac{T}{m\left(n+1\right)}\right)\left(1-\left(1-f\right)\gamma \right)+\frac{{\tilde{C_{h}}}_{\left(D\right)}T{\text{dar}}^{\alpha }}{m}\left(\frac{m}{n+1}+\frac{T}{2\left(2n+1\right)}-\frac{T^{2}}{2m\left(3n+1\right)}\right)\\ +\frac{{\tilde{C_{0}}}_{\left(D\right)}}{T}+\frac{n{\tilde{C}}_{\left(D\right)}I_{C}{\text{dar}}^{\alpha }}{m}\left[\frac{mT}{n+1}+\frac{T^{2}}{2\left(2n+1\right)}-\frac{T^{3}}{2m\left(3n+1\right)}-\frac{T_{d}\left(2m-T_{d}\right)}{2}\left(1+\frac{T}{m\left(n+1\right)}\right)\right.\\ \left.+\frac{nT_{d}^{1/n+1}}{T^{1/n}}\left(\frac{m}{\left(n+1\right)}-\frac{nT_{d}}{\left(2n+1\right)\left(n+1\right)}-\frac{T_{d}^{2}}{m\left(3n+1\right)\left(n+1\right)}\right)\right]-\frac{{\tilde{S}}_{\left(D\right)}I_{e}T_{d}^{1/n+1}{\text{dar}}^{\alpha }}{\left(n+1\right)T^{1/n}}. \end{array}$$

Next section finds the optimal value of equations (9) and (12)–(14) through the PSO technique.

## 4. Optimization of Proposed Inventory Model Using PSO

Particle swarm optimization is a nature-influenced optimization technique to solve the most complex optimization problems. The PSO developed by Kennedy and Eberhart [50] is influenced by the movement and intelligence of swarms. The swarms are considered as vector points in the domain space where the optimum value of a given objective function lies. There are four important vectors: (i) x-vector, which records the current position, (ii) p-vector that records the personal best, (iii) v- vector, which controls the velocity of the moving particle at each instance, and (iv) g-vector, which records the direction of the particle towards the global best position. The design of the algorithm is that the particles at each iteration move from its current position towards a new position by traveling to some extent parallel to its velocity vector, then to some extent towards its personal best, and to some extent towards the global best. The new position of the particle is the sum of these three vectors. The global best position is found by taking the best of all the personal best positions of all particles. The attributes are updated and proceeded for the next iteration. The process is repeated for a suitable number of iterations to achieve the best solution.

Four essential parameters control the movement of particles; a parameter to represent the coefficient of inertia “w” along with a damping inertial coefficient “wamp,” and two constants, *c*_1_ representing the acceleration of individual swarms, and *c*_2_ representing the social acceleration. These parameters are varied according to the choice of the optimization problem. To find the optimal total cost of the proposed inventory model, the algorithm of the variant of the PSO technique is presented as follows (see Algorithm 1).

## 5. Numerical Solution

Numerical example of the proposed inventory system is presented to support analytical derivation and discussion. Let us consider the proposed inventory system with the following setup: assume that the ordering cost is $750 per order, cost of raw material is $10 per unit, the cost to hold the item is $2.5 per item per unit time, and the selling price of each item is $18. Since the system allows both defective and perfect items, let the percentage of non-defective items be taken as 0.95, while the maximum lifetime of the item is considered as three years. Let the supplier admit a permissible delay of the payment period of two years for which the buyer earns 5% interest, where the interest charged per year in stocks by the supplier is taken as 8% per year. Let us further consider the parameter settings for the PSO: *C*_0_  =  $750, *C*=$10, *C*_*h*_=$2.5, *S*=$18, *m*=3 years, *T*_*d*_=1.5 years (Case 1), *T*_*d*_=1 year (Case 2), *I*_*e*_=0.05, *I*_*c*_=0.08, *n*=1, *d*=150, *r*=0.85, *γ*=0.5, *a*=1.2, and *α*=0.78.

Use the PSO for the inventory system to obtain the optimal solution of the proposed inventory model, as shown in Table 2.

### 5.1. Numerical Example Using Triangular Neutrosophic Set through PSO

A numerical example has been given to test the proposed inventory system using a triangular neutrosophic set to show the effect of the impreciseness of the cost parameters $\tilde{C_{h}}=\langle \left(0.5,1,1.5:0.5\right),\left(2.5,3,3.5:1\right),\left(3.5,4,4.5:0.5\right)\rangle$, $\tilde{C}=\langle \left(7.5,8,8.5:0.5\right),\left(8.5,9,9.5:1\right),\left(9.5,10,10.5:0.5\right)\rangle$, $\tilde{C_{0}}=\langle \left(599.5,600,600.5:0.5\right),\left(749.5,750,750.5:1\right),\left(849.5,850,850.5:0.5\right)\rangle$, $\tilde{S}=\langle \left(17.5,18,18.5:0.5\right),\left(19.5,20,20.5:1\right),\left(22.5,23,23.5:0.5\right)\rangle$. Thus, by de-neutrosophication of the triangular neutrosophic parameters, the values of the cost parameters are ${\tilde{C}}_{\left(D\right)}=\text{\$}9$, ${\tilde{C_{h}}}_{\left(D\right)}=\text{\$}2.6667$, ${\tilde{C_{0}}}_{\left(D\right)}=\text{\$}733.333$, ${\tilde{S}}_{\left(D\right)}=\text{\$}20.333$. For *f*=0.95, *m*=3 years, *T*_*d*_=1.5 years (Case 1), *T*_*d*_=1 year (Case 2), *I*_*e*_=0.05, *I*_*c*_=0.08, *n*=1, *d*=150, *r*=0.85, *γ*=0.5, *a*=1.2, and *α*=0.78. The optimal solution in the neutrosophic environment is shown in Table 2.

The PSO is implemented to find the optimal total inventory costs *TC*_1_, and *TC*_2_ with neutrosophic cost parameters along with the parameters of the proposed PSO algorithm *w*=1, wdamp=0.99, *c*_1_=2, *c*_2_=0.2. Table 2 shows a higher total cost at *n*=0.75 because the demand rate increases with time at 0 < *n* < 1, so the total quantity is higher and the total cost of inventory systems increases. The total cost is lower at *n*=2 because the demand rate decreases with time at *n* > 1; therefore, the total quantity is lower, and consequently, the total cost of the inventory systems decreases. Estimating the values of the cost parameters under actual market conditions is a big task for decision makers. The advantage of using neutrosophic numbers is that the decision maker can choose appropriate values for the cost parameters based on real market conditions. Neutrosophic numbers contain truth, hesitance, and falsity membership functions. These membership functions are helpful for establishing cost parameters to overcome impreciseness. Using neutrosophic numbers, the decision maker can choose appropriate values for cost parameters based on real market conditions.

By changing the values of *ϵ*_1_ and *ϵ*_2_ in the numerical example, the optimal solution was obtained as shown in Table 3. Both total inventory costs $\tilde{TC_{1}}$ and $\tilde{TC_{2}}$ increase when *ϵ*_1_ < *ϵ*_2_ and decrease when *ϵ*_1_ > *ϵ*_2_. If *ϵ*_1_ < *ϵ*_2_, then the cost parameters increase and the total cost also increases. If *ϵ*_1_ > *ϵ*_2_, then the values of the cost parameters decrease and the total cost also decreases. Here, *ϵ*_1_ and *ϵ*_2_ are the smallest positive constants between (0, 1) for the proposed inventory cost parameters. For all values of *ϵ*_1_ and *ϵ*_2_, the optimal inventory costs in the neutrosophic domain are lower than those in the crisp environment.

### 5.2. Sensitivity Analysis

Based on the numerical example of the inventory system shown in the previous section, a sensitivity analysis is performed by changing the parameters by a specific percentage, depending on the parameter boundary, based on the sensitivity analysis performed. For the sensitivity analysis, consider one parameter at a time and keep the other parameters fixed.

**Figure 2:**
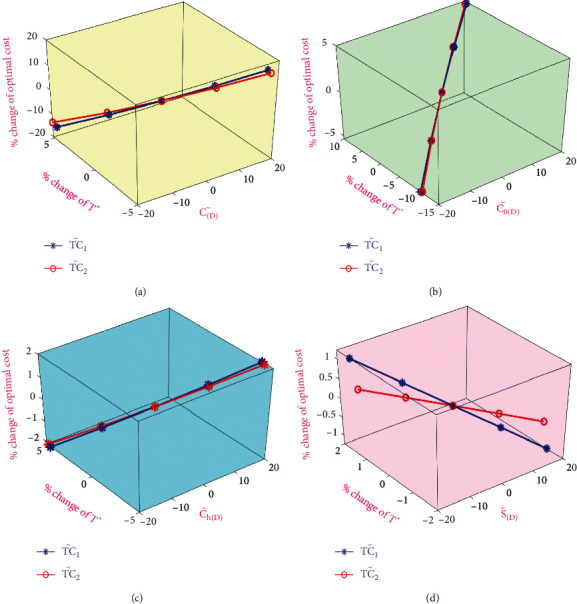
Sensitive analysis on optimal costs.

Table 4 shows that total inventory costs $\tilde{TC_{1}}$ and $\tilde{TC_{2}}$ are more sensitive to the purchase cost $\left({\tilde{C}}_{\left(D\right)}\right)$ and moderately sensitive to the holding cost $\left({\tilde{C_{h}}}_{\left(D\right)}\right)$ and the ordering cost $\left({\tilde{C_{0}}}_{\left(D\right)}\right)$ but less sensitive to the selling price $\left({\tilde{S}}_{\left(D\right)}\right)$. In both $\tilde{TC_{1}}$ and $\tilde{TC_{2}}$, the holding cost changes by 20%; if the holding cost increases, then $\tilde{TC_{1}}$ also increases in an analogous manner obtained from Figure 2. The total inventory costs $\tilde{TC_{1}}$ and $\tilde{TC_{2}}$ are less sensitive to the selling price, which can be seen in Figure 2. It is also observed that $\tilde{TC_{1}}$ and $\tilde{TC_{2}}$ are moderately sensitive to the ordering cost as shown in Figure 2. $\tilde{TC_{1}}$ and $\tilde{TC_{2}}$ are highly sensitive to the purchase cost, since the total cost is directly related to the purchase cost. Furthermore, when reliability is high, the price of buying an item dependent on reliability also increases.

**Figure 3:**
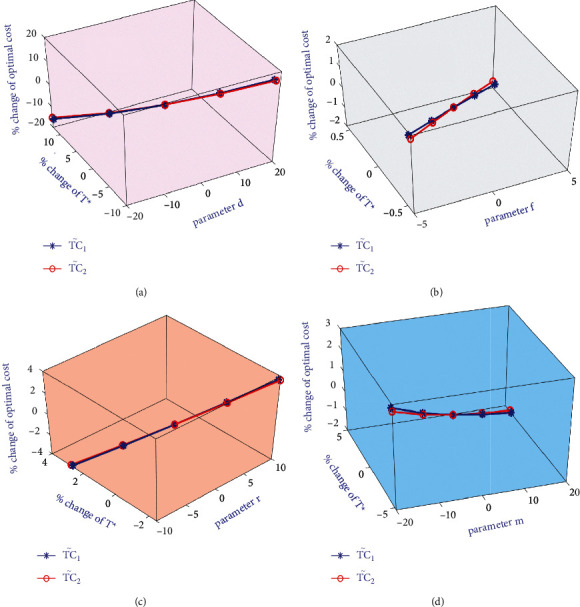
Sensitive analysis graphically on optimal costs vs other inventory related parameters.

Table 5 shows that the total costs $\tilde{TC_{1}}$ and $\tilde{TC_{2}}$ are highly sensitive to *d* and moderately sensitive to *m*, f, and *r* but less sensitive to *I*_*e*_, *γ*, and *T*_*d*_. Both total costs $\tilde{TC_{1}}$ and $\tilde{TC_{2}}$ are highly sensitive to *d*; according to credit concepts, from a practical point of view, if the parameter *d* increases, then total costs also increase, as shown in Figure 3. Both $\tilde{TC_{1}}$ and $\tilde{TC_{2}}$ are moderately sensitive to *m*, f, and *r*, which is graphically shown in Figure 3. The parameter *f* is increased as well decreased by 5% and 2%, because if a percentage of *f* increases by more than that value of *f* exceeds its permissible value, which shows that $\tilde{TC_{1}}$ and $\tilde{TC_{2}}$ decrease a limited amount but not very much, if non-defective items of inventory increase. The total optimal costs $\tilde{TC_{1}}$ and $\tilde{TC_{2}}$ of the proposed inventory model decrease with a lower rate, as shown in Figure 3 if the parameters *T*_*d*_ and *I*_*e*_ increase.

### 5.3. Managerial Implications

The value of any product is generally time-dependent. The cost of several products will increase as time progresses and the same will decrease for a few specific products. Most food items are perishable for which the value of such products will decrease, prolonging the storage period. Like wine, the cost of a few select products will increase when the storage period increases. This research convinced decision makers that perishable products should be stored in a healthier environment to lengthen their shelf life and reduce their rate of deterioration.

From a managerial standpoint, this study contributes to a better understanding of the reliability of the product. A well-maintained product will have higher demand, resulting in higher profits during sales. Furthermore, the managerial fact is that in order to keep such a product, management may have to incur higher holding costs. This means that we must balance these factors so that our total inventory cost is kept to a minimum.

The efficiency of deteriorating items, the cost patterns, and the reliability factors have been protectively represented in the flow of the inventory system. Therefore, this research can be widely applied in managing the inventory system where the reliability and deteriorating rates are concerned. Consequently, the current inventory model was created to depict these realistic features that may be utilized to forecast the system's elements.

## 6. Conclusion and Future Direction

The proposed inventory systems consist of various essential and complex functions to deal with the ordering and storing of products in which there are several uncertainties. Finding a generalized optimum order quantity model with uncertainty is a challenging task. In this article, an optimum order quantity inventory model without shortages in determining the items with reliability, the time-dependent power pattern, and the allowed time delay. This improves the inventory system, addresses the nature of items for time-dependent deterioration factors, and the cost pattern imprecise for a real scenario. The key advantage of this model is that it covers many sorts of product demand rates. The uncertainty in the cost pattern is dealt with the concept of impreciseness using triangular neutrosophic number. This study discussed and formulated the inventory model with reliability-dependent demand and analyzed the solutions by altering various parameter settings. A modified PSO algorithm is presented that fits the inventory model, and the same is implemented to find the optimal total cost in two different scenarios relating to time delay. PSO is utilized in both the crisp and neutrosophic senses, and the neutrosophic environment in which the solution is obtained is analyzed by sensitivity analysis. The proposed inventory system can be developed multiitems with different maximum lifetimes for items. The inventory system under a probabilistic or possibilistic environment with a different form of uncertainty such as intuitionistic fuzzy, interval type-2 fuzzy, Hesitant fuzzy sets, etc. The main limitation of this model is that it cannot allow more than 10% of defective items. Shortages are not allowed in this model, but shortages generally occur in the inventory system. This is the drawback of this model, so it can further develop this model under shortages.

## Figures and Tables

**Figure 4:**
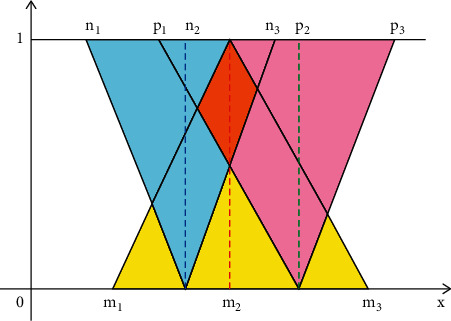
Graph of triangular single-valued neutrosophic number.

**Algorithm 1:**
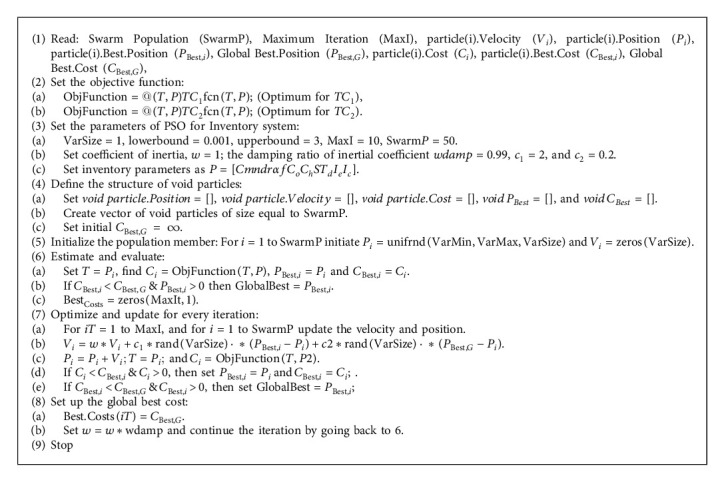
Proposed PSO algorithm for the inventory model.

**Table 1:** Contribution-based comparison of this article with earlier literature.

Articles	Demand rate	Deterioration	Imprecise parameters	Nature of impreciseness	Defective items	Discount on defective items	Delay in payment
Wee et al. [38]	Constant	No	No	No	Yes	No	No
Khanra et al. [39]	Quadratic	Constant	No	No	No	No	Yes
Chen and Teng [40]	Constant	Product's maximum life time	No	No	No	No	Yes
Pal et al. [41]	Ramp type	Weibull	Cost parameters and inflation rate	Triangular fuzzy	No	No	No
Wu et al. [42]	Constant	Product's maximum life time	No	No	No	No	No
San-Jose et al. [43]	Power pattern of time	No	No	No	No	No	No
Mahapatra et al. [44]	Price, stock, reliability, and advertisement	Weibull	Cost parameters	Triangular fuzzy	No	No	Yes
Mullai and Surya [45]	Constant	No	Cost parameters	Triangular neutrosophic	No	No	No
Sepehri et al. [46]	Price	Constant	No	No	Yes	No	No
Karakatsoulis and Skouri [47]	Constant	No	No	No	Yes	No	No
Luo et al. [48]	Constant	No	No	No	Yes	No	No
Present paper	Reliability and power pattern of time	Product's maximum life time	Cost parameters	Triangular neutrosophic	Yes	Yes	Yes

**Table 2:** Optimal inventory costs in different environments.

*n* value	Environment	*T* ^*∗*^	*TC*_1_(*T*^*∗*^)	*Q* ^*∗*^	*T* ^*∗*^	*TC*_2_(*T*^*∗*^)	*Q* ^*∗*^
1	Crisp	1.12183	2805.97	207.348	1.10380	2800.32	206.823
0.75	Neutrosophic	1.05876	2649.95	209.915	1.01887	2736.37	208.858
1	Neutrosophic	1.11206	2585.17	207.063	1.11793	2669.15	207.234
2	Neutrosophic	1.27544	2420.30	199.442	1.38407	2479.68	201.550

**Table 3:** Optimal variation of inventory costs under neutrosophic domain.

Values of *∈*_1_ and *∈*_2_	*T* ^*∗*^ in years	$\tilde{TC_{1}}\left(T^{*}\right)$	*T* ^*∗*^ in years	$\tilde{TC_{2}}\left(T^{*}\right)$
*∈*_1_=0.1, *∈*_2_=0.9	1.08924	2644.80	1.09369	2699.11
*∈*_1_=0.3, *∈*_2_=0.7	1.10047	2614.64	1.10560	2728.92
*∈*_1_=*∈*_2_=0.5	1.11206	2585.17	1.11793	2669.15
*∈*_1_=0.7, *∈*_2_=0.3	1.12405	2555.54	1.13070	2639.04
*∈*_1_=0.9, *∈*_2_=0.1	1.13644	2525.77	1.14394	2608.75

**Table 4:** Sensitivity analysis for the different inventory related costs.

Parameter	% change	*T* ^*∗*^ (years)	$\tilde{TC_{1}}\left(T^{*}\right)$	% change in $\tilde{TC_{1}}\left(T^{*}\right)$	*T* ^*∗*^ (years)	$\tilde{TC_{2}}\left(T^{*}\right)$	% change in $\tilde{TC_{2}}\left(T^{*}\right)$
${\tilde{C}}_{\left(D\right)}$	−20	1.16292	2220.50	−14.11	1.17547	2303.83	−13.69
	−10	1.13663	2403.16	−7.04	1.14539	2486.88	−6.83
	10	1.08903	2766.57	7.02	1.09274	2850.73	6.80
	20	1.06737	2947.40	14.01	1.06952	3031.67	13.58
	−20	0.99567	2445.99	−5.38	0.99543	2532.48	−5.12

${\tilde{C_{0}}}_{\left(D\right)}$	−10	1.05546	2517.50	−2.62	1.05843	2601.76	−2.52
	10	1.16596	2649.55	2.49	1.17448	2733.13	2.40
	20	1.21753	2711.08	4.87	1.22851	2794.17	4.68
	−20	1.12811	2616.88	1.23	1.13118	2684.18	0.56

${\tilde{S}}_{\left(D\right)}$	−10	1.12000	2601.06	0.61	1.12458	2676.68	0.28
	10	1.10429	2569.21	−0.62	1.11125	2661.59	−0.28
	20	1.09669	2553.22	−1.24	1.10452	2653.97	−0.57
	−20	1.16250	2530.31	−2.12	1.17168	2613.93	−2.07

${\tilde{C_{h}}}_{\left(D\right)}$	−10	1.13642	2558.39	−1.04	1.14383	2641.88	−1.02
	10	1.08925	2611.68	1.03	1.09376	2695.80	1.00
	20	1.06782	2637.63	2.03	1.07114	2721.84	1.97

**Table 5:** Sensitivity analysis for the different inventory related parameters.

Parameter	% change	*T* ^*∗*^ (years)	$\tilde{TC_{1}}\left(T^{*}\right)$	% change in $\tilde{TC_{1}}\left(T^{*}\right)$	*T* ^*∗*^ (years)	$\tilde{TC_{2}}\left(T^{*}\right)$	% change in $\tilde{TC_{2}}\left(T^{*}\right)$
*d*	−20	1.24254	2192.72	−15.18	1.25469	2285.96	−15.37
	−10	1.17180	2390.87	−7.52	1.18060	2466.05	−7.61
	10	1.06073	2776.18	7.39	1.06398	2868.85	7.48
	20	1.01599	2964.36	14.67	1.01686	3065.58	14.85

*γ*	−20	1.11085	2594.48	0.36	1.11664	2678.48	0.35
	−10	1.11145	2589.82	0.18	1.11728	2673.82	0.17
	10	1.11267	2580.51	−0.18	1.11858	2664.49	−0.17
	20	1.11328	2575.85	−0.36	1.11923	2659.83	−0.35

*m*	−20	1.05589	2657.17	2.79	1.05896	2741.42	2.71
	−10	1.08583	2617.74	1.26	1.09042	2701.87	1.23
	10	1.13527	2557.98	−1.05	1.14226	2641.61	−1.03
	20	1.15598	2534.37	−1.97	1.16398	2618.08	−1.91

*T*_*d*_	−20	1.11206	2635.78	1.96	1.12545	2700.67	1.18
	−10	1.11206	2610.47	0.98	1.12214	2685.13	0.60
	10	1.11206	2559.86	−0.98	1.11276	2652.65	−0.62
	20	1.11206	2534.55	−1.96	1.10653	2635.53	−1.30

*I*_*e*_	−20	1.12811	2616.88	1.23	1.13118	2684.16	0.56
	−10	1.12000	2601.06	0.61	1.12458	2676.68	0.28
	10	1.10429	2569.21	−0.62	1.11125	2661.59	−0.28
	20	1.09669	2553.18	−1.24	1.10452	2653.97	−0.57

*r*	−10	1.14135	2485.90	−3.84	1.14885	2565.39	−3.89
	−5	1.12631	2536.30	−1.89	1.13288	2618.07	−1.91
	5	1.09868	2632.62	1.84	1.10388	2718.77	1.86
	10	1.08608	2678.78	3.62	1.09064	2767.02	3.67

*f*	−5	1.10570	2634.19	1.90	1.11118	2718.21	1.84
	−2	1.10958	2604.18	0.74	1.11530	2688.18	0.71
	2	1.11446	2566.89	−0.71	1.12048	2650.87	−0.68
	5	1.11791	2540.78	−1.72	1.12414	2624.73	−1.66

## Data Availability

No data were used to support this study.

## Appendix

Definition 1 A.1.
*Single-valued neutrosophic set: “a single-valued neutrosophic set* $\left(\tilde{S}\right)$ *of a single-valued independent variable (x) is defined by* $\tilde{S}$ *=*  $\left\{\langle x;\left[\pi_{\;\tilde{S}}\left(x\right),\theta_{\tilde{S}}\left(x\right),\eta_{\;\tilde{S}}\left(x\right)\right]\rangle :x\in X\right\}$*, where*$\pi_{\;\tilde{S}}\left(x\right),\theta_{\;\tilde{S}}\left(x\right),\eta_{\;\tilde{S}}\left(x\right)$*represent the concept of truth, hesitation*, *and falsity membership functions, respectively.”* Neutro-normal: let us consider three points *p*, q, and *r* for which $\pi_{\;\tilde{S}}\left(p\right)=1,\theta_{\;\tilde{S}}\left(q\right)=1,\eta_{\;\tilde{S}}\left(r\right)=1$; then, $\tilde{S}$ is defined as neutro-normal.

Definition 2 A.2.
*Neutro-convex: “a neutrosophic set* $\tilde{S}$ *is called neutro-convex if the following condition holds: (i)* $\pi_{\tilde{S}}\left(\lambda \alpha +\left(1-\lambda \right)\beta \right)\geq \min\left(\pi_{\;\tilde{S}}\left(\alpha \right),\pi_{\;\tilde{S}}\left(\beta \right)\right)$ *, (ii)* $\theta_{\;\tilde{S}}\left(\lambda \alpha +\left(1-\lambda \right)\beta \right)\leq \max\left(\theta_{\;\tilde{S}}\left(\alpha \right),\theta_{\;\tilde{S}}\left(\beta \right)\right)$ *, and (iii)* $\eta_{\;\tilde{S}}\left(\lambda \alpha +\left(1-\lambda \right)\beta \right)\leq \max\left(\eta_{\;\tilde{S}}\left(\alpha \right),\eta_{\;\tilde{S}}\left(\beta \right)\right)$ *, whereα*, *β* ∈ *R, andλ* ∈ [0,1]*.”*

Definition 3 A.3.
*Triangular single-valued neutrosophic number: “a triangular single-valued neutrosophic number* $\left(\tilde{S}\right)$ *is defined as* $\tilde{S}=\langle \left(m_{1},m_{2},m_{3};n_{1},n_{2},n_{3};p_{1},p_{2},p_{3}\right.\rangle$ *. Here,* $\pi_{\;\tilde{S}}:R⟶\left[0,1\right]$ *is the truth membership function,* $\theta_{\;\tilde{S}}:R⟶\left[0,1\right]$ *is the hesitation membership function, and the falsity membership function is* $\eta_{\;\tilde{S}}:R⟶\left[0,1\right]$ *, where the membership functions are mathematically and graphically (* Figure 4) *defined as follows.”*

(A.1) $$\begin{array}{c} \pi_{\tilde{S}}\left(x\right)=\left\{\begin{array}{cc} x-m_{1}/m_{2}-m_{1}, & \text{for}m_{1}\leq x<m_{2}\\ 1, & \text{for}x=m_{2}\\ m_{3}-x/m_{3}-m_{2}, & \text{for}m_{2}<x\leq m_{3}\\ 0, & \text{otherwise} \end{array}\right.\\ \theta_{\tilde{S}}\left(x\right)=\left\{\begin{array}{cc} n_{2}-x/n_{2}-n_{1}, & \text{for }n_{1}\leq x<n_{2}\\ 0, & \text{for }x=n_{2}\\ x-n_{2}/n_{3}-n_{2}, & \text{for }n_{2}<x\leq n_{3}\\ 1, & \text{otherwise} \end{array}\right.\\ \eta_{\tilde{S}}\left(x\right)=\left\{\begin{array}{cc} p_{2}-x/p_{2}-p_{1}, & \text{for }p_{1}\leq x<p_{2}\\ 0, & \text{for }x=p_{2}\\ x-p_{2}/p_{3}-p_{2}, & \text{for }p_{2}<x\leq p_{3}\\ 1, & \text{otherwise} \end{array}\right.. \end{array}$$

De-neutrosophication of triangular single-valued neutrosophic number: use the removal area technique to evaluate the de-neutrosophication value of triangular single-valued neutrosophic number $\tilde{S}=\langle \left(m_{1},m_{2},m_{3}:\mu \right),\left(n_{1},n_{2},n_{3}:\nu \right),\left(p_{1},p_{2},p_{3}:\zeta \right)\rangle$. The de-neutrosophic form of $\tilde{S}$ is given by $\tilde{S_{D}}$  =  1/12(*m*_1_+2*m*_2_+*m*_3_+*n*_1_+2*n*_2_+*n*_3_+*p*_1_+2*p*_2_+*p*_3_).
